# Public Hygiene in America

**Published:** 1878-03

**Authors:** 


*Public Hygiene in America:* Being the Centennial Discourse Delivered before the International Medical Congress, Philadelphia, Sept. 1876. By HENRY J. BOWDITCH, M. D. with Extracts from Correspondence from the various States. Together with a Digest of American Sanitary Law, by HENRY G. PICKERING, Esq. Boston: Little Brown & Co. London: Truebner & Co. 1877.

To one interested in sanitary science this is an exceedingly valuable volume In Hygiene there is no higher authority in America than Dr. Bowditch

Anything from his pen must claim attention not only from physicians but from the educated world The Centennial Address has already by vote of the Congress been printed in an abridged form and sent to the Governors of these States, and to those of the Provinces of Canada.

The Appendix, which comprises the larger part of the volume, is made up of extracts from letters from every part of the Union, written by physicians and others, in reply to a list of questions, framed by the author, in the interest of Public Hygiene and State or Preventive Medicine. It contains also the law of Soil Moisture as a Cause of Consumption in Massachusetts and elsewhere. It is a book which should be widely and carefully read, and its suggestions acted upon with a hearty good will and unanimity, which alone can secure the condition of high Public Health foreshadowed in the author's thought.

